# Untangling Tau: Molecular Insights into Neuroinflammation, Pathophysiology, and Emerging Immunotherapies

**DOI:** 10.3390/cimb45110553

**Published:** 2023-11-02

**Authors:** Ryder Davidson, Reese I. Krider, Philip Borsellino, Keith Noorda, George Alhwayek, Thomas A. Vida

**Affiliations:** Kirk Kerkorian School of Medicine at UNLV, 625 Shadow Lane, Las Vegas, NV 89106, USA; davidr6@unlv.nevada.edu (R.D.); krider1@unlv.nevada.edu (R.I.K.); borsep1@unlv.nevada.edu (P.B.); noordk2@unlv.nevada.edu (K.N.); alhwag1@unlv.nevada.edu (G.A.)

**Keywords:** tau protein, neuroinflammation, Alzheimer’s disease, amyloid-beta peptide, protein aggregation, hyperphosphorylation, immunotherapy, monoclonal antibody

## Abstract

Neuroinflammation, a core pathological feature observed in several neurodegenerative diseases, including Alzheimer’s disease (AD), is rapidly gaining attention as a target in understanding the molecular underpinnings of these disorders. Glial cells, endothelial cells, peripheral immune cells, and astrocytes produce a variety of pro-inflammatory mediators that exacerbate the disease progression. Additionally, microglial cells play a complex role in AD, facilitating the clearance of pathological amyloid-beta peptide (Aβ) plaques and aggregates of the tau protein. Tau proteins, traditionally associated with microtubule stabilization, have come under intense scrutiny for their perturbed roles in neurodegenerative conditions. In this narrative review, we focus on recent advances from molecular insights that have revealed aberrant tau post-translational modifications, such as phosphorylation and acetylation, serving as pathological hallmarks. These modifications also trigger the activation of CNS-resident immune cells, such as microglia and astrocytes substantially contributing to neuroinflammation. This intricate relationship between tau pathologies and neuroinflammation fosters a cascading impact on neural pathophysiology. Furthermore, understanding the molecular mechanisms underpinning tau’s influence on neuroinflammation presents a frontier for the development of innovative immunotherapies. Neurodegenerative diseases have been relatively intractable to conventional pharmacology using small molecules. We further comprehensively document the many alternative approaches using immunotherapy targeting tau pathological epitopes and structures with a wide array of antibodies. Clinical trials are discussed using these therapeutic approaches, which have both promising and disappointing outcomes. Future directions for tau immunotherapies may include combining treatments with Aβ immunotherapy, which may result in more significant clinical outcomes for neurodegenerative diseases.

## 1. Introduction

Alzheimer’s disease (AD) is the most common form of dementia [1]. Occurring predominantly in the population after 60 years old, 4.5 million individuals over the age of 65 years live with clinical AD with prevalence doubling essentially every 10 years after 65 years old [2,3]. Moreover, this disease is projected to only become more common. The estimated prevalence of AD is estimated to increase to 13.8 million by 2060 [4]. More highly associated as an acquired disease, the most prominent acquired risk factors occur in a person’s midlife age which include developing: hypertension, dyslipidemia, cerebrovascular disease, artery atherosclerosis, type 2 diabetes, and traumatic brain injury [5]. Hypertension and diabetes are closely interlinked due to similar risk factors such as endothelial dysfunction, vascular inflammation, arterial remodeling, atherosclerosis, dyslipidemia, and obesity [6]. Chronic inflammation, a commonality between these conditions, has been increasingly linked with AD, suggesting that inflammation may influence individual susceptibility to the disease [7]. Additionally, insulin resistance and deficiency, commonly seen in type 2 diabetes, can interact with amyloid-β protein and tau protein phosphorylation, leading to the onset and development of AD [8]. Dyslipidemia, characterized by abnormal lipid levels in the blood, can contribute to atherosclerosis, a condition where fatty deposits build up in arteries, potentially leading to cerebrovascular diseases. Cerebrovascular diseases can compromise blood flow to the brain, which is a known risk factor for AD. Traumatic brain injury (TBI) has been shown to result in cognitive deficits and has been linked to chronic neurodegeneration, including AD [9]. TBI-induced injuries can accelerate amyloid β (Aβ) production and accumulation, arterial stiffness, and tau hyperphosphorylation, all of which are associated with AD [10]. Essentially, an aging population is at greater risk as these risk factors become more common in the general population. A genetic predisposition occurs towards early-onset AD with mutations in amyloid precursor protein (APP) and presenilin (PSEN1/2) genes, but late-onset genetic risk factors include allelic variation in apolipoprotein E (Apo E) [11]. Although a rarer cause of AD, the symptoms tend to be more acute with a similar pathology.

AD is a clinical diagnosis given in the context of symptom progression and includes significant economic burdens. These symptoms include in the early stages of the disease deficits in some executive functions and short-term memory and in the late stages of the disease deficits in language, long-term memory, and behavioral changes [12,13]. Together, this leads to significant impairment in daily life and eventually requires dedicated caretakers. The estimated economic burden due to AD is more than 11 million family members and other unpaid caregivers provided an estimated 16 billion hours of care to people with Alzheimer’s or other dementias in 2021. Unpaid dementia caregiving was valued at $271.6 billion in 2021 [4]. The average per-person Medicare payments for services to beneficiaries aged 65 and older with AD or other dementias are almost three times as great as payments for beneficiaries without these conditions [14]. The total payments in 2023 for health care, long-term care, and hospice services for people aged 65 and older with dementia are estimated to be $345 billion [14], but this does not factor in the emotional toll paid by the patients and their caretakers. Increasing concern about understanding the causes and pathology of AD has grown as the annual number of deaths from 2000 to 2019 by 145%, now is the 6th leading cause of death in all adults in the US [14]. Two pathologies associated with AD are beta-amyloid plaques (Aβ) and tau bundles. In this review, we explore the role that tauopathies play in the development of AD and current medical therapies targeting this pathology.

## 2. Role of Neuroinflammation in the Progression of Alzheimer’s Disease

In recent decades, the molecular understanding of AD has evolved considerably. Historically, AD research primarily centered on the accumulation of Aβ plaques and neurofibrillary tangles (NFTs) as the principal pathological hallmarks. However, advancements in neuroimmunology and neuroimaging have identified neuroinflammation as an additional core pathological feature of AD [15].

The brain’s innate immune response is an essential defense mechanism, activated in the presence of harmful stimuli, including infections, toxins, or physical injuries. This acute neuroinflammatory response initiates a cascade of events aimed at tissue repair and restoration within the brain [16]. However, challenges arise when an imbalance occurs in pro-inflammatory and anti-inflammatory signaling. Such an imbalance can precipitate a state of chronic neuroinflammation, characterized by the persistent activation of glial cells, predominantly microglia and astrocytes. This state produces a sustained release of cytotoxic molecules, including pro-inflammatory cytokines such as TNF-α, IL-1β, and IL-6, reactive oxygen species (ROS), and nitric oxide (NO). This prolonged inflammatory condition adversely affects neuronal function and is implicated in the initiation and progression of AD [17].

Microglia, often referred to as the brain’s resident macrophages, exhibit a complex role in AD. Their activation in the disease’s early stages facilitates the phagocytosis and clearance of pathological Aβ and aggregates of the tau protein. This protective role of microglia has been demonstrated in various animal models, suggesting their potential to mitigate early AD-associated pathologies. However, with prolonged activation, the protective mechanisms of microglia become compromised, leading to reduced neurotoxin clearance, increased Aβ accumulation, tau aggregate propagation, and neuronal loss [18,19].

Astrocytes, another predominant glial cell type, undergo significant transformations in AD. Under physiological conditions, astrocytes support neuronal function, provide essential nutrients, and maintain cerebral homeostasis. However, in AD, they transition into a state termed “reactive astrogliosis”. Reactive astrocytes, identifiable by their morphological changes and increased expression of glial fibrillary acidic protein (GFAP), interact extensively with Aβ plaques. While this interaction aims to isolate the plaques from surrounding neurons, prolonged exposure to Aβ induces astrocytes to release pro-inflammatory mediators. Moreover, their role in Aβ clearance becomes compromised, further exacerbating the disease’s progression. Recent findings also indicate that astrocytes may contribute to tau hyperphosphorylation, a critical event in NFT formation [20,21].

The relationship between neuroinflammation and AD, while recognized for several years, remains a topic of intensive research. The main question is whether neuroinflammation acts as an antecedent, predisposing the brain to AD, or emerges because of the disease’s progression. Preliminary evidence suggests early microglial activation in AD, potentially precedes significant Aβ accumulation. As the disease advances, both microglia and astrocytes appear to transition from a neuroprotective to a neurodegenerative phenotype. This evolving paradigm underscores the potential role of neuroinflammation in both the onset and progression of AD [16].

### 2.1. Pathophysiology of Tauopathies

The accumulation of aggregates composed of the tau protein characterize a class of neurodegenerative disorders called tauopathies [22]. The primary function of tau is to stabilize microtubules in neuronal cells [23]. Tau is inherently disordered without a defined secondary or tertiary structure and prone to many post-translational modifications (PTMs) [24,25,26,27,28,29]. Mutations in the gene that encodes tau, *MAPT*, alternative splicing, PTMs, or some combination of the three can promote the aggregation of monomers of tau into oligomers, straight filaments, and NFTs [25]. These aggregates, along with β-amyloid aggregates, are believed to play a vital role in the pathogenesis of AD [30].

The *MAPT* gene is found on chromosome 17 in humans. The gene consists of 16 exons in which alternative splicing produces primarily six isoforms of tau through alternative splicing of the gene. The differences in each of these isoforms primarily lie in the presence or absence of exons 2, 3, and 10. Exon 10 encodes a microtubule-binding site that is functionally similar to but sequentially unique from 3 other exons in the *MAPT* gene. Thus, the 6 isoforms of tau, though mutually exclusive, are often split into two categories: those lacking exon 10/with 3 microtubule binding sites (3R) and those including exon 10/with 4 microtubule binding sites (4R) [31].

During human development, the 3R isoforms dominate. But once adulthood is reached, the 4R isoform has increased until equal amounts of 4R and 3R are present in neurons. However, this equal ratio is altered in many neurodegenerative tauopathies, particularly AD. In AD, the 4R:3R fluctuates as the disease progresses. Early on in disease progression, the 4R form tends to dominate, either due to increases in 4R or decreases in 3R, while 3R becomes the predominant isoform in the later stages of disease progression [32,33,34]. Interestingly, no structural differences occur among pure 4R tau fibrils, pure 3R tau fibrils, or mixed 4R and 3R tau fibrils. Regardless, the differential expression and aggregation of these isoforms are believed to play a key role in tau-related neurodegeneration in AD [32]. Evidence exists that suggests the 3R isoform becomes the preferential component to incorporate tau aggregates as disease severity increases [35]. The reasons and mechanism for this are unclear, but the structural differences in the 4R and 3R isoforms are believed to play a vital role in the pathogenesis of tauopathies and thus are the focus of several immunotherapies.

**Figure 1:**
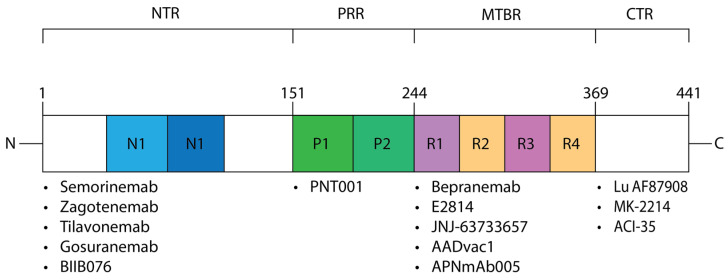
The tau protein domains mapped to antibody-based therapies. The major regions of the tau protein are depicted. The associated regions are listed with their residue count including the N-terminal region (NTR), proline-rich region (PRR), microtubule binding region (MTBR), and the C-terminal region (CTR). Immunotherapies that interact with these major domains are listed below. Note an incongruence exists with the residues listed for the MTBR described for Bepranemab and JNJ-63733657, which bind to 235–250 and 217 respectively, but are still categorized as an anti-MTBR immunotherapy.

Tau isoforms exhibit variations in length and size, with the longest known isoform in the human central nervous system (CNS) comprising 441 amino acids, with 80 Ser or Thr residues, 56 negative (Asp or Glu) residues, 58 positive (Lys or Arg) residues and 8 aromatic (5 Tyr and 3 Phe, but no Trp) residues [36]. With this amino acid composition, the native tau protein is highly soluble, charged, and hydrophilic. However, mutations in the *MAPT* gene along with an array of post-translational modifications are believed to be pivotal in facilitating its transformation into non-soluble aggregates. Tau can be categorized into four primary regions: the N-terminal region (NTR), the proline-rich region (PRR), the microtubule-binding region (MTBR), and the carboxy-terminal region (CTR, Figure 1).

The NTR spans amino acids 1–151 in the longest isoform and is characterized by its non-microtubule binding properties. This region exhibits considerable variability across different tau isoforms and is implicated in interactions with non-microtubule cellular components, including the plasma membrane, endoplasmic reticulum, and Golgi apparatus. These interactions link various cellular components to microtubules and are believed to facilitate intracellular signaling and transport pathways involving tau [24]. Specifically, the NTR is known to associate with membrane-associated proteins such as annexins and Ca^2+^-regulated membrane-binding proteins [37]. It possesses acidic properties and maintains a highly negative charge at physiological pH. The NTR, along with the PRR, constitutes the most intrinsically disordered regions of tau, which is believed to contribute to the pathogenesis of tau [29,31,37].

Located between amino acids 151–244 in the longest isoform, the PRR is basic and positively charged at physiological pH. This region is a key hotspot for post-translational modifications, particularly phosphorylation, acetylation, and glycosylation [28], which are pivotal for tau’s functionality and are detailed below [37,38,39]. The modifications of these residues, along with those within MTBR, affect tau’s ability to bind microtubules. Modifications along other regions of tau can affect functionality, but they do not affect microtubule binding to the same degree [36,40,41,42]. 

The MTBR encompasses amino acids 244–369 in the longest isoform and is primarily responsible for tau’s interaction with microtubules. This region is composed of 3–4 repeats of 31–32 residues [29]. Unlike the PRR and CTR, the MTBR is less susceptible to post-translational modifications. However, modifications in adjacent regions are thought to influence the MTBR’s microtubule-binding capability and its propensity for pathological aggregation. This region contains either three or four distinct binding domains and is also basic and positively charged at physiological pH. Because the 4R isoforms have an extra one of these binding domains compared to the 3R isoforms, they show a higher affinity to bind microtubules [43].

The CTR spans amino acids 369–441 in the longest isoform and is the least variable among tau isoforms. This conservation suggests the presence of key residues essential for maintaining tau’s functional integrity. These conserved residues tend to be serine and lysine, which are amino acids prone to modification [25]. Behind the PRR, the CTR undergoes the most post-translational modifications, and these modifications are believed to be key in regulating tau’s interaction with microtubules [37,44]. 

### 2.2. Tau Basic Function

The normal functions of tau are well characterized and have identified an array of functions, but the degree of physiological impact for these functions is still unclear [31]. Mainly localized to neurons, and to a lesser extent oligodendrocytes and astrocytes [45], tau’s most prominent known function is its association with neuronal microtubules, particularly its assembly and stabilization of these microtubules [46,47,48]. It also plays roles in cell membrane interactions [49], gene expression [50], and DNA conformational changes and stabilization [51,52].

### 2.3. Tau Aggregation

The pathophysiological mechanisms underlying tau aggregation remain poorly understood. Historically, large insoluble aggregates of tau proteins, commonly known as NFTs, were considered the primary cause in the pathogenesis of tau-associated disorders. However, emerging evidence has challenged this notion. For instance, Lasagna-Reeves first identified the pathogenic role of tau oligomers in 2012, highlighting their involvement in the formation of pretangles, neuritic plaques, and neurophil threads. Intriguingly, some studies have posited that NFTs may even serve as a protective mechanism against neuronal death in tauopathies [53] and this form of tau has even been observed in healthy brains [54].

Recent studies have increasingly focused on the pathogenic capabilities of tau oligomers, which serve as precursor aggregates to NFTs. These oligomers form through the assembly of post-translationally modified tau monomers, emphasizing the crucial role of post-translational modifications in oligomer formation. They have been implicated in potentiating neuronal damage and subsequent neurodegeneration [55,56,57,58]. Structurally, tau oligomers gain order and adopt a secondary β-sheet conformation, primarily composed of highly phosphorylated or pathologically truncated tau proteins [59].

While the toxicity of various tau forms is still under investigation, current evidence suggests that non-toxic forms likely include monomers, straight filaments, paired helical filaments, NFTs, and ghost tangles [60]. In contrast, toxic forms are more likely to be dimers/trimers, small oligomers (300–500 kDa), and granular tau oligomers (approximately 1800 kDa) [61,62,63,64].

Animal studies have further exemplified the role of tau oligomers in neurodegeneration. Mice injected with tau oligomers exhibited more severe cognitive and neuronal abnormalities compared to those injected with other tau forms like NFTs [65,66]. Moreover, tau oligomers have a stronger propensity to induce tau misfolding and aggregation in unaffected neuronal regions compared to other tau forms [67]. This suggests that NFTs may not be as central of an element in the neurotoxic cascade as once thought and that research for effective treatments for tauopathies might lie in specifically targeting these oligomeric forms.

## 3. Tau Post-Translational Modifications

As many as 35% of residues in tau are involved in varying post-translational modifications [24], which include serine, threonine, tyrosine, lysine, arginine, asparagine, histidine, and cysteine. The most prominent and known post-translational modifications that are key to the pathophysiology of tau are phosphorylation and acetylation. However, a large array of other modifications exists, including but not limited to ubiquitination, SUMOylation, methylation, glycation, and proteolytic processing [24,25,26,27,28,29].

### 3.1. Phosphorylation

Phosphorylation is the most well-known and potentially the most pathogenically significant post-translational modification (PTM) in tau-associated disorders, playing a pivotal role in both normal function and pathological developments associated with tau proteins [24]. Tau proteins have 85 potential phosphorylation sites, comprising 45 serine, 35 threonine, and 5 tyrosine, which demonstrate the modification’s importance as over half of tau’s residues that can be post-translationally modified are believed to be phosphorylated [29,68,69].

In the context of normal cellular processes, phosphorylation facilitates a range of functions. However, when inappropriate or excessive, a phenomenon known as hyperphosphorylation, can disrupt tau’s interactions with microtubules and enhance its tendency to form disease-causing aggregates [70]. This alteration in tau can compromise cytoskeletal integrity, and hinder axonal transport, synapse function, and cell signaling, a disruption well-documented in various diseases including Alzheimer’s [24,70,71].

The biological function of tau protein hinges on the meticulous regulation of its phosphorylation status, a process coordinated through a complex interplay between various enzymes responsible for adding or removing phosphate groups. Kinases such as GSK3β, cdk5, and CK1 add phosphate groups to tau, while phosphatases like PP1 and PP2A, predominantly undertake the removal of these groups, with PP2A being notably central in tau dephosphorylation in the human brain [72,73,74]. This intricate system also encompasses feedback mechanisms, with phosphatases influencing signaling pathways like the ERK1/2 MAPK cascade, which subsequently activates GSK3β [75]. Moreover, tyrosine kinases, including members of the SRC and ABL families, target specific amino acid residues associated with tau aggregation and Alzheimer’s disease, further illustrating complexity of the regulation for this amino acid post-translational modification [76,77].

A significant portion of phosphorylation sites reside in the microtubule-binding region (MTBR), yet other sites external to this region, such as Thr205, Ser396, and Ser404, are suspected to induce tau’s pathological conformation [74]. Additionally, certain missense mutations, that change proline 301 to a modifiable serine destabilize tau and enhance its propensity to aggregate, thereby altering its conformation significantly [78]. The phosphorylation at these sites can modulate tau’s interaction with microtubules (MT), influencing MT formation and potentially inhibiting microtubule interactions, preventing assembly or destabilization [79,80]. Notably, the phosphorylation of residues like Thr231 and Ser262 can directly inhibit microtubule interactions and destabilize microtubules [80].

Studies have pinpointed specific phosphorylated residues that either promote or inhibit tau aggregation. Residues such as Thr175, Ser202, Thr205, Thr212, and Ser422 foster aggregation, while others including S214, S258, S262, S293, S305, S324, and S356 inhibit the process [74,81,82,83,84,85,86,87]. Interestingly, tau demonstrates 3–4 times more phosphorylated residues in its aggregated state compared to its stable form, highlighting the critical role phosphorylation plays in tau’s pathological function [88].

In the later stages of AD, particularly in Braak stages V/VI (discussed below), a marked elevation occurs in the levels of phosphorylated residues such as Thr231, Ser202, Ser422, and Thr205 [28]. These phosphorylation dynamics not only influence tau’s regulatory role over microtubules but also affect its inherent stability and functionality. Consequently, these phosphorylated residues have emerged as focal points in the development of immunotherapies targeting tau’s aggregation and its disease-causing properties, targeting efforts towards mitigating the adverse effects of tau phosphorylation in AD.

### 3.2. Acetylation

Acetylation is also a significant post-translational modification of tau. It has a substantial role in both the normal and pathological behaviors of tau in tauopathies, coming second only to phosphorylation in terms of research focus [89,90]. Acetyl groups are added mainly to lysine residues, a process that neutralizes their positive charge and thereby affects tau’s overall folding and interaction with other proteins.

Tau protein contains a rich sequence of lysine residues, offering numerous sites for acetylation. These lysines are also susceptible to other PTMs including ubiquitination, SUMOylation, methylation, and glycation, indicating a complex interplay of modifications that occur at these sites [24]. The acetylation process is intricately regulated by a set of enzymes, namely the P300/CBP complex acetylase, which facilitates acetylation, and HDAC6 and SIRT1 are central to the deacetylation process, targeting specific regions of tau for modification [24,89,91,92,93]. Despite their crucial roles, the exact functions of these enzymes in tauopathies are yet to be fully understood.

The acetylation landscape in AD displays a dysregulated balance in the activities of HDAC6 and SIRT1; the former exhibits increased activity while the latter is diminished, pointing to a complex role of acetylation in neurodegenerative conditions [92,94]. Fluctuating levels of histone deacetylases and histone acetyltransferases in neurodegenerative diseases further echo this complexity, hinting at the multifaceted role of acetylation in these disorders [92,94,95].

The specific acetylated residues on tau significantly impact its microtubule binding properties. Predominantly found in the microtubule-binding region (MTBR), these lysine sites, when acetylated, exhibit altered affinities for microtubules. For example, acetylation at K280 diminishes the region’s affinity for microtubules, whereas acetylation at other sites like K174 enhances tau’s tendency to aggregate instead [93,96,97]. Conversely, acetylation at residues such as K259, K290, K321, and K353 causes a reduction in tau aggregation [89].

Connections exist between varying levels of tau acetylation and different stages of AD, with heightened acetylation levels being noted in both early and moderate Braak stages of tauopathy and heavy acetylation linked to late-stage AD [93,98]. Moreover, acetylation at K280 has emerged as a more disease-specific marker compared to phosphorylated tau in tauopathies, presenting a nuanced understanding of tau’s role in disease pathology [96]. The diverse acetylation patterns observed in AD brains as opposed to healthy controls further underscore the potential significance of individual acetylated residues in the disease process [93,96,99].

The K321 and S324 sites potentially act as an acetylation-phosphorylation switch. For example, when K321 is acetylated, S324 tends to remain unphosphorylated and vice versa, giving insight into one of tau’s regulatory mechanisms [100]. Despite this understanding of acetylation and disease progression, most research on therapeutic intervention for tauopathies predominantly targets tau phosphorylation. However, further investigation of tau acetylation could reveal a promising pathway for future therapeutic interventions, encouraging a deeper exploration into the roles of acetylation in tauopathies.

## 4. Pathophysiologic Mechanisms

The pathophysiological mechanisms of tauopathies that ultimately lead to neuronal death have been described to follow two different, intertwined models: the gain-of-function and loss-of-function models [101,102,103].

### 4.1. Gain-of-Function Model

The gain-of-function model describes processes that lead to neuronal death from aberrant tau forms. In the gain-of-function model, tau proteins acquire new toxic properties through various mutations to the *MAPT* gene and through the various post-translational modifications described above. These modifications lead to an altered tau protein that can foster a series of pathological cascades including the formation of NFTs. The pathological tau can propagate in a prion-like manner, leveraging cellular structures such as exosomes for intercellular transmission, leading to widespread neurodegeneration. This model delineates the multifaceted pathological processes including mitochondrial dysfunction and neuroinflammation, orchestrated by the aberrant tau, which disrupts cellular homeostasis and fosters a neurodegenerative environment [102,103,104].

### 4.2. Loss-of-Function Model

The loss-of-function model emphasizes the detrimental effects on normal processes stemming from the loss of normal tau function. This model focuses on diseased tau’s loss of normal regulation of microtubule stabilization, axonal transport, and neuronal integrity. In tauopathies, the mutated or misfolded tau loses its ability to bind to microtubules, resulting in microtubule destabilization and impaired axonal transport, which is pivotal for neuronal function. Consequently, neurons face structural instability and a disruption in the transport of essential nutrients and organelles, paving the way for neurodegeneration. This model underscores the necessity of understanding the physiological roles of tau, as restoring the normal function of tau could potentially emerge as a therapeutic strategy in treating tauopathies [101,103,104].

### 4.3. Tau Propagation

The propagation of tau follows a well-documented and predictable pattern in the brain, often categorized using Braak staging, which encompasses six stages (I–VI) to delineate the progression of the disease [105,106]. Initially, mild to severe alterations take place in the transentorhinal layer (stages I–II), which escalate to significant affection in the limbic stages, involving both the transentorhinal region and the entorhinal cortex, with slight involvement of the first Ammon’s horn sector (stages III–IV). This progression eventually leads to the widespread destruction of isocortical association areas in the final stages (stages V–VI).

Tau propagation operates through a series of intricate mechanisms, with the most popular hypothesis being the trans-synaptic spread of tau between neurons via a prion-like mechanism. This process explains the progression of tauopathies across different brain regions [107,108,109] but a consensus does not exist in this mechanism. Although tau NFTs can result in the propagation of pathological forms of tau [110], tau oligomers, along with causing neuronal death, are the key conformations that facilitate its spread throughout the brain [111,112,113,114]. These oligomers may facilitate spread through a seeding mechanism, where tau aggregates induce the misfolding of native tau proteins, as a pivotal process in fostering the spread of tau pathology [111,115]. Key players in this mechanism include the “central region” of tau, with features of the PRR and MTBR, notably residues 224–230, and the mid-domain, particularly the HVPGG epitope, residues 299–303, which may play crucial roles in the cell-to-cell propagation and seeding of tau aggregates [116,117]. Moreover, the microtubule-binding region (MTBR) remains central to tau’s primary function and its role in aggregation and propagation, with nerve cell processes and oligodendrocytes being primary players in the induction of tau filaments [107,117].

Unique to tau propagation, compared to other prion-like diseases, is the intracellular location of tau aggregates [107]. As tau accumulates, it leads to cellular dysfunction and ultimately cell death, releasing tau oligomers into the extracellular space through various mechanisms including neuronal death and secretion by living neurons through exocytosis or direct release in its naked form [118,119,120]. These oligomers can aggregate into paired helical filaments known as “ghost tangles”, a phenomenon well documented in the late stages of AD and can be taken up via endocytosis [121,122]. Extracellular tau and β-amyloid foster tau release through a positive feed-forward regulation, with vesicle secretion potentially playing a role in regulating intracellular tau levels [119,123]. The unconventional secretion of tau involves various mechanisms such as direct translocation across the plasma membrane, release through secretory lysosomes, microvesicle shedding, and vesicle-mediated exosome release, influenced by factors including calcium-dependent neuronal activity and phosphorylation states [119,124,125,126,127,128,129]. Tau can also be released through nanotubes, filamentous cell membrane structures that bridge cellular connections, which can link the cytoplasmic compartments of adjacent cells [130,131].

Transgenic mouse brain models have been instrumental in elucidating the dynamics of tau transmission and spreading in AD. The htau mouse model has revealed the age-dependent trajectory of tau pathology, emphasizing the progressive nature of tau-associated cognitive and synaptic dysfunctions [132]. In vivo studies have spotlighted the presence of tau in both cerebrospinal and interstitial fluids of diseased mice, and the age-dependent decreases in brain interstitial fluid monomeric tau levels in certain human tau transgenic mice [133], suggesting a mechanism of tau aggregate propagation involving the formation and sequestration of more monomeric tau as the disease progresses [134]. Synthetic tau fibrils have demonstrated the capacity to induce and propagate tau pathology, highlighting the critical role of specific tau conformations in disease progression and supporting the hypothesis of active induction spread of tau through nearby cells via seeding and exocytosis, as opposed to more passive diffusion of the aggregates throughout distant regions of the brain [135,136]. Insights from a Selenica mouse model have revealed the potential long-term impacts of brief elevations in Aβ production on tauopathy risk, affirming the consensus that AD initially manifests as a β-amyloid pathology, followed by tauopathy after several years [15,136].

## 5. Therapeutic Interventions

Both passive and active immunotherapies are being developed and are classified under three major categories: (1) the targeted epitopes of tau; (2) specific conformations of tau aggregates; (3) phosphorylated amino acids on native or pathologic tau, or linear amino acid sequences on tau (most commonly at the N-terminal domain or the MTBR). All the listed immunotherapies, no matter the target, aim to act against the prion theory of tauopathies. Although we have arranged immunotherapies into different themes, some immunotherapies crossover due to the complex nature of antibody functions. Notably, experimental mice did not have concurrent Aβ pathology, a noted limitation for the generalizability of these treatments to AD pathology.

### 5.1. Conformation Specific Epitopes

#### 5.1.1. APNmAB005

APNmAB005 is an IgG that specifically targets synaptic tau oligomers, uniquely found in early-stage AD aggregates rather than late-stage [136]. More specifically, its epitope is a conformation found in aggregations of both 3R and 4R tau isoforms outside of fibrillar tau’s Beta-rich core. In vitro, it was found to be most selective for oligomers, partially reactive against fibrils, and barely reactive against monomers. Furthermore, this drug has shown immunoreactivity in human brain samples. Both nerve and glial cells demonstrated reactions against esotau, a term coined for early-stage oligomers in various tauopathies. The presence of esotau and its association with AP-NmAB005 has prompted questions regarding the possibility of a shared pathophysiology across different tauopathies. In rTg4510 mice, which express the P301L tau variant, the drug neutralized toxic tau aggregates. While the drug neutralized these toxic aggregates, it did not seem to clear them. Nevertheless, this action was associated with increased neuronal survival and the preservation of synaptic integrity [136].

#### 5.1.2. Zagotenemab

Zagotenemab is developed based on MCI-1, (also called LY3303560) [137], an antibody that recognizes a distinctive conformational epitope that is closely associated with early pathologic tau [138]. For this epitope to be identified, a specific set of criteria needs to be met. It requires the presence of amino acids 7–9 and another set of amino acids situated within the range of 312–314. This identification is further contingent on multiple tau proteins interacting with one another. Moreover, a particular folding mechanism is needed to bring these two regions into close contact. It is crucial to note that the presence of this epitope is significantly attenuated with a deletion in the amino acid region spanning from 46 to 241. Despite the three required conditions for binding, the primary epitope is at the NTD [139]. The selectivity of MCI-1 and subsequently termed Zagotenemab, for pathological tau was the driving force for its development.

### 5.2. Phosphoepitopes

#### 5.2.1. JNJ-63733657

JNJ-63733657 is an IgG1 antibody against MTBR w/high affinity for tau phosphorylated at residue 217, aiming to prevent aggregation and action of tau seeds [140]. Currently in phase 2 study for mild AD, suggesting some merit to the claim of success in pre-clinical mouse models.

#### 5.2.2. Lu AF87908

Lu AF87908 is an IgG1 that targets hyperphosphorylated tau, focusing on the phosphorylation site serine-396 (pS396-tau) [141,142]. This specific site is crucial as it is directly associated with disease progression as seeding activity is correlated with the amount of pS396-tau and tau aggregates. The pS396-tau is present in both intracellular and extracellular tauopathies. The drug works by depleting the hyperphosphorylated and aggregated tau in a manner that is proportional to the amount of tau aggregation. In rTg4510 mice models, the hyperphosphorylated tau leads to aggregation, which consequently results in forebrain neuronal death. Interestingly, Lu AF87908 binds to tau tangle structures, especially in the cortex and hippocampus. This binding remains stable at 3- and 14-days post-dose. However, a decline in this binding occurs by the 21st day. Furthermore, at 4 days after dosing, a dose-dependent reduction occurs in pS396-tau: a decrease of 36% at 60 mg/kg and 48% at 120 mg/kg.

#### 5.2.3. MK-2214

MK-2214, AKA Ta1505, an IgG2a antibody against pSer413, formed from GSK3Beta activity. pSer413 was chosen as it has been found within many structures associated with early disease AD in the CA1 region of the hippocampus, including neurons even without NFTs [143]. Tested on tau609 and tau704 mice, which show memory impairment and early tau modifications at 6 months of age improved memory compared to controls. Control antibodies included Ta4 (IgG2b against pSer396) and Ta9 (IgG3 against pSer396). Additional findings in mouse models included decreased tau hyperphosphorylation, oligomerization, synapse loss, tangle formation, and neuronal loss.

#### 5.2.4. PNT001

PNT001 is a drug that targets cis-ptau, which is the phosphorylated Thr231-Pro, a motif in tau [144]. This motif can either be in its trans (normal) form or cis (pathologic) configuration. This cis-ptau is found in the early stages of AD and chronic traumatic encephalopathy (CTE). Notably, in traumatic brain injury (TBI) mice models, neurons predominantly produce cis-ptau within hours. This results in cistauosis, leading to widespread neuronal degeneration and brain atrophy [145]. Binding cis-ptau inside cells targets it for proteasomal degradation while binding it outside cells prevents the spread of tauopathy. The trans-ptau is crucial as it promotes vital microtubule functions in neurons. In contrast, the cis-ptau disrupts these functions, causing complications. The isomerase, Pin1, plays a role in converting cis-ptau to trans-ptau. However, the function of this protein is impaired in conditions like AD and TBI. Intriguingly, both AD and CTE exhibit a similar tau isoform profile and phosphorylation state. This similarity has been observed in individuals with AD and boxers diagnosed with CTE, suggesting they might have similar pathogenic mechanisms [146,147].

#### 5.2.5. ACI-35

ACI-35, or more currently ACI-35.030, is a liposomal (MPLA) vaccine developed to target the amino acids 393–408, with a particular emphasis on pS396 and pS404 [148,149,150]. The foundation for this targeting is based on the recurring theme of phosphorylation at sites pS396, pS400, and pS404. Notably, these sites undergo phosphorylation by GSK3 Kinases during pathological conditions. Furthermore, under CD spectroscopy, the epitope reflects a β-pleated sheet conformation.

Preclinical testing used P301L mice and wild-type (WT) mice. At the chosen observational period, P301L mice display early clinical signs of tauopathy, manifesting mainly as mild cognitive impairments. The vaccination schedule was as follows: the first dose was administered at 6 months, initiating the observation period. Subsequent doses were given on days 0, 7, and 14, with the last two doses spaced monthly, on days 56 and 84. The animals were eventually sacrificed at 9 months.

In terms of outcomes, an immune response was evident in both mouse strains after two injections (days 0 and 14), with blood sampling on day 28 for WT mice and day 21 for P301L mice. Importantly, this immune response displayed selectivity for the phosphorylated epitope, with only a marginal immune response against the corresponding non-phosphorylated sequence. A noticeable decrease in soluble pS396 tau occurred in the brainstem and forebrain. Interestingly, levels of pS404 and T231 tau remained unaffected. Additionally, a reduction was observed in insoluble pS396 tau in the forebrain. Vaccinated P301L mice exhibited reduced weight loss, indicative of improved health or reduced disease severity. Furthermore, the vaccine influenced a specific behavioral metric known as ‘clasping’, serving as an indicator of significant motor impairment in mice. Vaccinated P301L mice experienced a delay in the onset of clasping, and fewer vaccinated mice exhibited clasping by the end of observation. However, vaccination did not bring about any improvements in overall motor performance.

### 5.3. Linear Amino Acid Epitopes: N-Terminal Domain

#### 5.3.1. Semorinemab

Semorinemab is a humanized IgG4 antibody that targets the N-terminal domain of tau, specifically amino acids 6–23 [151,152]. This particular region was chosen for its potential to bind to tau irrespective of its phosphorylation pattern to sequester and inhibit the extracellular spread of tau. Notably, three specific phosphorylation sites have been highlighted for their significant seeding activity: Thr231, Ser235, and Ser262 [153]. In tauP301-L-Tg mice, which is specifically designed to manifest pathology from the 2N4R tau variant. In these mice, Semorinemab significantly reduced pathological tau accumulation when compared to a control IgG2a (similar to full effector IgG1 in humans) [151,152]. Furthermore, in vitro experiments using neurons co-cultured with microglia revealed differences between antibody isotypes [154]. When exposed to the full effector IgG1, the protective function of this antibody was lost. However, both IgG4 and attenuated IgG1 retained their efficacy. This observed loss in protection is believed to be a consequence of harmful effector functionality when it interacts with active, dysregulated microglia. This adverse interaction is speculated to be due to an escalated cytokine release from microglia [154].

#### 5.3.2. Tilavonemab

Tilavonemab, also referred to as 8E12 or HJ8.5, underwent a transformation from its original form as an IgG2b to become an IgG4 antibody. It specifically targets the N-terminal domain (NTD) of tau, zoning in on amino acids 25–30 [155]. In experimental P301S mice, Tilavonemab displayed promising therapeutic outcomes over a span of three months [156]. Not only did it attenuate brain loss and bolster sensorimotor functions, but it also effectively reduced the levels of phosphorylated tau. Furthermore, a notable improvement was observed in the associative learning capabilities of the treated mice. One of the standout attributes of Tilavonemab is its selectivity. The drug is adept at blocking the uptake of misfolded tau derived from brain lysates, emphasizing its potential in targeting extracellular tau aggregates [156]. 

#### 5.3.3. Gosuranemab

Gosurenamab is an IgG4 antibody that specifically targets the N-terminal domain (NTD) of tau, binding to amino acids 15–24 [123]. This antibody shows an affinity for both the secreted extracellular tau (etau) and the full-length tau (0N4R). In vitro studies using primary human cortical neurons revealed neutralizing etau resulted in lowered levels of Aβ. Conversely, introducing etau to the neuronal environment led to an increase in Aβ levels. Moreover, in vivo studies demonstrated that etau induces a decrease in sAPPalpha (soluble amyloid precursor protein-alpha), which is a neuroprotective protein. However, antibodies targeting etau had the opposite effect, causing an increase in sAPPalpha levels. The epitope was designed to target the N-terminal fragments, which are present in the interstitial fluid and play a pivotal role in the progression of tauopathies [157]. These fragments are thought to expedite the spread of pathologic tau and induce a state of hyperexcitability in neurons.

#### 5.3.4. BIIB076

BIIB076, also known as 6C5, is an IgG1 antibody that targets the mid-domain of tau, specifically binding to amino acids 125–131 [158]. In vitro, BIIB076 blocks both the uptake and aggregation of tau, even after the uptake process has commenced. Moreover, it was able to halt the propagation within interneurons. BIIB076 binds monomeric tau, pre-formed fibrillar tau, and even tau derived from the human brains of both healthy individuals and those with AD [159].

### 5.4. Linear Amino Acid Epitopes: Microtubule-Binding Region

#### 5.4.1. Bepranemab

Bepranemab is an IgG4 antibody against amino acids 235–250, near the microtubule-binding domain due to the idea that this region is more important for pathogenesis than other regions, namely the N-terminal region [116]. Other regions, including both terminal domains of tau, can be cleaved, creating a truncated yet still pathogenic tau, adding another benefit for targeting the center. Pre-clinical measures of efficacy were shown through AT8 (an antibody against pS202-pT205-pS208 to label tau lesions) immunoreactivity. The mice genotypes were THY-tau30, which express human 1N4R protein with P301S and G272V amino acid mutations, and HtauP301L, expressing human 2N4R tau protein with P301L mutation. Injection of pathogenic fibrils that do not have the epitope for Bepranemab (recombinant P301L-K18 fibers) revealed the ability to block further transmission from acting on secreted tau from neurons affected with the fibrils. Since AT8 also cannot bind the injected fibril, decreased AT8 immunoreactivity in the contralateral hemisphere could not be from acting on the recombinant fibrils, further confirming the purported mechanism of Bepranemab. 

#### 5.4.2. E2814

E2814 is an IgG1 antibody against MTBR of tau [117]. It is bi-epitopic for MTBR 4 and mono-epitopic for MTBR 3, binding specifically to an HVPGG motif, which is found more often in AD brain than in normal brain, although it is not selective for pathological tau variations. The epitope is 19 amino acids located in the ends of both R2 and R3 of the MTBR. Specifically, it is the combined _299_HVPGGGS_305_ and PHF6 (_306_VQIVYK_311_) sequences, forming an aggregative seeding motif. The PHF6 (_306_VQIVYK_311_) and PHF6* (not targeted in this antibody) sequences are vital if not necessary for pathogenesis, playing a role in seeding and aggregation due to their propensity for forming β-pleated sheets helps explain why. In vitro, E2814 inhibited tau aggregation in vitro. In vivo, it attenuated deposition of Sarkosyl-insoluble tau (pathogenic tau fibrils are insoluble in Sarkosyl detergent), correlating to decreased fibril aggregation.

#### 5.4.3. AADvac1

AADvac1 is a vaccine designed around a peptide sequence, specifically 294KDNIKHVPGGGS305, with an added N-terminal cysteine that facilitates its attachment to its carrier protein, the keyhole limpet hemocyanin (KLH) [160]. Notably, this peptide contains the HVPGG sequence from the MTBR region, specifically found in MTBR2 and MTBR4. Previous research by the same authors introduced a monoclonal antibody (DC8E8) against a slightly altered version of this sequence, emphasizing HVPGGG rather than just HVPGG [161]. Identified as a crucial player in tau oligomerization, the HVPGG(G) epitope discriminates between regular and pathological tau. These specific regions become exposed due to either C-terminal folding or removal when tau enters a pathological state. Additionally, this region was bound in early pathological structures. Subsequently, these findings suggest that tau proteins might not adopt a pathological state without rendering the MTBR region accessible. 

AD-SHR7 rats were used for preclinical studies [160,162]. The immunization regimen for these rats was as follows: they received their first dose at 2 months of age, followed by another dose three weeks later, with the subsequent three doses administered monthly. After reaching 6.5 months of age, the rats were sacrificed for serum analysis. The immunoglobulins (IGs) generated post-immunization retained their selectivity for pathological tau, preferring the vaccine’s peptide sequence over physiological tau. The variety of IGs produced included IgG1, IgG2a, IgG2b, IgG2c, and IgM. The elevated production of IgG1 suggested a high affinity with a robust Th2 response. Additionally, the treated rats demonstrated a reduction in several pathological markers such as a decrease in Sarcosyl-insoluble tau, reduced highly phosphorylated tau across all lengths, and a lowered presence of tau oligomers/polymers. Furthermore, the immunized group exhibited enhanced performance in sensorimotor tests. Additionally, these IGs are effectively recognized and bound to neurofibril lesions sourced from human AD brains in vitro. All of these immunotherapies are summarized in Table 1.

## 6. Clinical Trials

### 6.1. Safety and Tolerance

Both passive and active therapies have shown to be relatively well tolerated. Regarding the active immunotherapies, no serious adverse effects have been uncovered in phase I or phase II safety clinical trials. The AADvac1, consisting of 6 total injections, is immunogenic without any serious adverse events outside of injection site reactions and confusion [163,163]. Additionally, ACI-35 has shown a similar safety profile with no serious adverse events [149].

The monoclonal antibodies aimed at the N-terminal regions of tau with passive immunotherapy have a proven safety profile and are generally only related with mild to moderate adverse events even at higher doses. Zagotenemab, Tilavonemab, and Gosuranemab show no serious or severe adverse events related to treatment [156]. Semorinemab was associated with mild bruising and pain at the injection site, but no serious dose-limiting adverse effects.

More recent monoclonal antibodies that target the central tau domain, microtubule binding domain, or the cis isomer of tau showed mixed safety profiles. PNT001, which targets the cis isomer of tau, the drug was well tolerated at all doses tested in phase 1 [164]. Bepranemab, which binds to the microtubule-binding domain, showed no adverse effects in phase I [165]. The two-microtubule-binding domain monoclonal antibodies, E2814 and JNJ-63733657 likewise showed no severe adverse effects. Nausea, headache, and vomiting were reported with E2814 [166] and back pain and headache reported with JNJ-63733657 [140]. BIIB076, which binds to the central domain of tau had some associated signs of toxicity. During the phase I trial, the higher dose ranges were shown to have dose-limiting toxicity, specifically headaches, dizziness, vomiting, nausea, and hypotension [167]. This could potentially be related to the lower specificity of BIIB076 for pathologic tau contributing to disruption of physiologic tau in vivo.

Overall, the safety of tau immunotherapy in aggregate has been demonstrated in both phase I and II clinical trials across different diverse arrays of molecular targets, with the one exception being BIIB076. This suggests that effective tau immunotherapy would likely be safe to administer.

### 6.2. Effectiveness at Achieving Disease-Oriented and Clinical Endpoints

The current track record of tau immunotherapy in phase II clinical trials is mixed with regard to meeting patient and disease-oriented endpoints. The disease-oriented outcomes suggest that tau immunotherapies have been effective at attacking their molecular targets; however, with respect to clinical endpoints, the results have been largely negative.

Current monoclonal antibodies aimed at targeting the N-terminal region of tau have failed to reach their primary endpoints. The Gosuranemab phase II clinical trial in AD patients with mild cognitive impairment failed to reach its primary endpoint, which was significant changes to the clinical dementia rating, and sum of boxes (CDR-SB) score compared to placebo. Likewise, the drug also failed to meet secondary endpoints such as reduction in the Alzheimer’s Disease Assessment Scale-Cognitive Subscale (ADAS-Cog13), the Alzheimer Disease Cooperative Study Activity of Daily Living (ADCS-ADL), the Mini-Mental State Examination (MMSE) and the Functional Assessment Questionnaire (FAQ) [168]. Later that year, it was also discovered that treatment with Gosuranemab lead to worsened ADAS-Cog13 scores [167]. In the TAURIEL phase II study, Semorinemab likewise failed to meet primary and secondary endpoints which included significant decreases in CDR-SB scores, ADAS-Cog13, and ADCS-ADL respectively [169]. Semorinemab also failed to slow pathologic NFT accumulation in comparison to placebo on MRI [164]. The ongoing LAURIET phase II study of Semorinemab showed 43.6% slowing of decline on the ADAS-Cog11, though no other clinical benefit [170]. The phase II clinical trial examining Tilavonemab did not result in significant decreases in CDR-SB, secondary outcome measures such as ADAS-Cog14, RBANS, MMSE, FAQ, and ADCS-ADL, nor led to protection from brain atrophy or neurofilament light (NfL) [167]. Zagotenemab phase II clinical trial failed to meet the primary endpoint which included a significant reduction in integrated Alzheimer’s Disease Rating Scale (iADRS) [171].

Evidence for the effectiveness of active immunotherapy targeting tau also exists. Phase II clinical trial, ADAMANT, of the AADvac1 immunization against pathologic tau proteins, showed both disease-oriented and clinically measurable differences between placebo. AADvac-1 was immunogenic and produced an IgG response in 95% of immunized patients. Blood NfL rose more slowly in AADvac1 population vs. placebo, and a significant reduction in pathologic tau variants. Regarding clinical endpoints, a slower decline occurs in CDR-SB, MMSE, and ADL among AD patients in the younger age range. White matter density also increased in treatment groups compared to control groups [162].

### 6.3. Potential Reasons for N-Terminal Tau Monoclonal Antibody Ineffectiveness

A challenge that is faced when attempting to design passive immunotherapy to hyperphosphorylated tau is difficulty reaching the target. The propagation of pathologic tau species generally occurs intracellularly. Previous studies showed that levels of tau in the CSF and levels of pTyr181 tau are significantly increased in patients with AD, though are absent in other tau-mediated neurodegenerative disorders [172]. This ultimately suggests that extracellular tau is likely not involved in the pathogenesis of tau-mediated neurodegeneration and that any treatment aimed at targeting the tau protein would need to target the intracellular component. Likewise, previous literature highlights the importance of exosomal propagation and the use of tunneling nanotubes in the propagation of neurodegenerative mediators such as the tau protein [131]. It is possible that the use of monoclonal antibodies may be less optimal for targeting tau-mediated pathology. Intracellular agents that can stop NFT production or propagation through exosomal or tunneling nanotubes may prove to be a more effective therapy.

Another potential reason offered for the ineffectiveness of current N-terminal tau monoclonal antibodies includes the low diversity of targeted epitopes. Many of the N-terminal antibodies bind multiple tau species, though it could be possible that the pathologic species is not covered by these drugs. One possible explanation offered is that truncation of the N-terminal region of tau would lower the potential target diversity of these monoclonal antibodies. Interest is increasing in developing tau monoclonal antibodies that bind to the core regions and the microtubule-binding domains of the tau protein as they play a role in the spreading of tau pathology [173]. The effectiveness of these therapies has yet to be ascertained.

The apparent ineffectiveness of N-terminal tau immunotherapy could also be related to the complexity of AD pathogenesis and pathophysiology [174]. Previous studies showed a connection between NFT count and cognitive status in neurodegenerative disease even when compared to other proposed mediators such as Aβ [175]. However, it is possible that tau is not the principal mediator of neurodegeneration. Other mechanisms such as Aβ aggregation, neuroinflammation, free radical damage, and dysfunction of cholinergic neurons contribute to the pathogenesis of Alzheimer’s disease, and all these factors working in concert with one another may be responsible for advanced disease progression. It is likely that a multitargeted approach may be the most appropriate in tackling AD rather than relying upon a specific target.

## 7. Future Directions

Currently, the FDA has approved two Aβ directed immunotherapies- Aducanumab and Lecanemab. Individually, this is due to aducanumab showing a significant effect on brain Aβ removal, a reasonable likelihood of meaningful clinical benefit, and early Phase IV evidence that this benefit is seen in a subsequent trial; aducanumab received its FDA approval in 2020 [176,177]. Lecanemab in its Phase III trials showed a significant reduction in the decline of cognitive function and reduction in Aβ protein compared to placebo [178]. Both medications require further testing to determine long-term clinical efficacy.

Greater strides in treatment have been achieved in Aβ therapies due to the diversity of targets trialed with these medications, often focusing on different epitopes and forms of the Aβ such as monomers and oligomers, or disrupting the production, aggregation, or augmenting the degradation of Aβ [179]. A potential future direction with tau therapies is to similarly develop treatments targeting more stages of the tau bundle formation process. Similarly, increasing the efficacy of therapy delivery could be a future branch for exploring immuno-tau therapies. Much like the Aβ immunotherapy gantenerumab, which failed to achieve significant results in its Phase III trials, a new version called Trontinemab has shown promise of greater success due to its ability to combine with a medication that binds to transferrin receptors of endothelial cells of the blood-brain barrier, thus allowing for increased endocytosis of gantenerumab [180].

Lastly, one more future direction that can be trialed on tau immunotherapies with an acceptable side-effect profile is combining treatments with Aβ immunotherapy, the most beneficial being either Lecanemab or Aducanumab at this time. Both Aβ and tau proteins are associated with AD and are correlated with AD progression [30]. Combining medications to target both proteins may result in more significant clinical outcomes.

## Figures and Tables

**Table 1:** Immunotherapies Directed at the Tau Protein.

Drug Name or Identifier	Immunity Type	Drug Target	Stage of Development	NCT/jRCT	Drug Sponsor	MoA Category	Residues
APNmAb005	passive	synaptic oligomeric tau in early AD; aggregations of both 3R and 4R from misfolding	Stage I	NCT05344989	Aprinoia Therapeutics	Conformation	N/A
Bepranemab	passive	microtubule binding domain	Stage II	NCT04867616	Hoffmann-La Roche, UCB S.A.	Linear: MTBR	235–250
E2814	passive	microtubule binding domain (HVPGG epitope: adjoined _299_HVPGGGS_305_ and PHF6 (_306_VQIVYK_311_))	Stage II/III	NCT05269394	Eisai Co., Ltd.	Linear: MTBR	299–305 & 306–311
JNJ-63733657	passive	microtubule binding domain	Stage II	NCT04619420, NCT05407818, NCT03689153, NCT03375697,	Janssen	Phosphoepitope	217
Lu AF87908	passive	hyperphosphorylated tau protein	Stage I	NCT04149860	Lundbeck	Phosphoepitope	394 & 404
MK-2214	passive	pSer-413	Stage I	NCT05466422, jRCT2031220627	Merck	Phosphoepitope	413
PNT001	passive	cis-isomer of tau (pT231)	Stage I	NCT04096287	Pinteon Therapeutics	Phosphoepitope	231
Semorinemab	passive	N-terminal domain (residues 6–23) in monomeric/oligomeric tau regardless of phosphorylated state	Stage II	NCT03828747, NCT03289143	AC Immune SA, Genentech, Hoffmann-La Roche	Linear: NTD	6–23
Zagotenemab	passive	primary epitope is N-terminal domain of early tau pathological conformation, based on MC1 (amino acids 7–9 + residues 312–314 + conformational change)	Discontinued	NCT03518073	Eli Lilly & Co.	Conformation	7–9 & 312–314
Tilavonemab	passive	N-terminal domain (residues 25–30) of misfolded tau from brain lysates (extracellular)	Discontinued	NCT03712787	AbbVie, C2N Diagnostics, LLC	Linear: NTD	25–30
Gosuranemab	passive	N terminal domain (residues 15–24) of extracellular tau; targeted specifically at etau fragments found in CSF, which often contain this epitope.	Discontinued	NCT03352557	Biogen, Bristol-Myers Squibb	Linear: NTD	15–24
BIIB076	passive	central domain of tau; residues 125–131	Discontinued	NCT03056729	Biogen, Eisai Co., Ltd., Neurimmune	Linear: NTD	125–131
AADvac1	active	_294_KDNIKHVPGGGS_305_ with an extra N-terminal cysteine to attach to its carrier protein, keyhole limpet hemocyanin (KLH)	Stage II	NCT02579252	Axon Neuroscience SE	Linear: MTBR	294–305
ACI-35	active	residues 393–408 with pS396 and pS404 (this sequence forms a β-pleated sheet structure)	Stage II	NCT04445831	AC Immune SA, Janssen	Phosphoepitope	393–408; 396 & 404

## Data Availability

No new data were created or analyzed in this study. Data sharing is not applicable to this article.
